# Treating Sleep Problems in Patients with Schizophrenia

**DOI:** 10.1017/S1352465815000430

**Published:** 2015-07-30

**Authors:** Felicity Waite, Elissa Myers, Allison G. Harvey, Colin A. Espie, Helen Startup, Bryony Sheaves, Daniel Freeman

**Affiliations:** University of Oxford, Warneford Hospital, UK; East London NHS Foundation Trust, UK; University of California, Berkeley, USA; Sleep and Circadian Neuroscience Institute, University of Oxford, UK; Sussex Partnership NHS Foundation Trust, and University of Oxford, UK; University of Oxford, Warneford Hospital, UK

**Keywords:** Psychosis, cognitive behavioural therapy, insomnia, delusions, hallucinations

## Abstract

**Background:** Sleep disturbance is increasingly recognized as a major problem for patients with schizophrenia but it is rarely the direct focus of treatment. The main recommended treatment for insomnia is cognitive behavioural therapy, which we have been evaluating for patients with current delusions and hallucinations in the context of non-affective psychosis. **Aims:** In this article we describe the lessons we have learned about clinical presentations of sleep problems in schizophrenia and the adaptations to intervention that we recommend for patients with current delusions and hallucinations. **Method:** Twelve factors that may particularly contribute to sleep problems in schizophrenia are identified. These include delusions and hallucinations interfering with sleep, attempts to use sleep as an escape from voices, circadian rhythm disruption, insufficient daytime activity, and fear of the bed, based upon past adverse experiences. Specific adaptations for psychological treatment related to each factor are described. **Conclusions:** Our experience is that patients want help to improve their sleep; sleep problems in schizophrenia should be treated with evidence-based interventions, and that the interventions may have the added benefit of lessening the psychotic experiences. A treatment technique hierarchy is proposed for ease of translation to clinical practice.

## Introduction

Sleep disturbance is a major problem for people with schizophrenia. Up to 80% of people with schizophrenia report symptoms of insomnia (Cohrs, 2008). Our own work has shown that over half of patients with persecutory delusions report moderate or severe insomnia (Freeman, Pugh, Vorontsova and Southgate, 2009). Patients report substantial negative consequences of sleep disturbance and a desire for treatment (Waite et al., in press). Cognitive behavioural therapy is an established, evidence based intervention for treating insomnia (CBT-I) (e.g. Harvey, 2002; Espie, 2006). However, its application to patients with schizophrenia has not been described. A case series by our group indicated that it is feasible to use CBT-I in patients with schizophrenia and that improvements in sleep and persecutory delusions may arise (Myers, Startup and Freeman, 2011). Recently, we have conducted a pilot randomized controlled trial (RCT) targeting sleep problems in 50 patients with delusions and hallucinations in the context of non-affective psychosis (Freeman et al., 2013). The outcome data show that CBT-I, suitably adapted for this population, leads to a large effect size reduction in insomnia (*d* = 1.9) (Freeman, Waite, et al., in press). In this paper we share our experience of applying CBT-I in schizophrenia.

### Intervention overview

Two processes interact to regulate sleep and wakefulness: the sleep/wake homeostasis and the body's internal clock or circadian rhythm (Borbély, 1982). Biological and psychological factors contribute to the onset and maintenance of sleep disturbance. CBT treatment techniques (summarized in Table 1) aim to address both types of factors.

**Table 1.** Key components of standard cognitive behavioural therapy for insomnia

Treatment strategy	Main approach
Assessment	Sleep diaries are used as a key feature of assessment and treatment to establish the pattern of sleep on a nightly basis. In addition, actigraphy may be used. Actigraphy is the measurement of movement via a small accelerometer device worn on the wrist that can be used to estimate sleep via periods of inactivity, in conjunction with sleep diaries.
Formulation	Formulation focuses on developing a shared understanding of the factors that led to the development and persistence of the sleep disturbance.
Psychoeducation	Psychoeducation includes learning key information regarding the process of sleep and the impact of poor sleep.
Sleep hygiene education	Sleep hygiene is a specific psychoeducational intervention. It focuses on addressing a number of environmental and behavioural factors that may influence sleep quality and quantity.
Stimulus control	The aim of stimulus control is to strengthen the association between the bed and good quality sleep. This is achieved by limiting the time in bed not sleeping. Therefore the bed is used only for sleeping and sexual activity. One is instructed to only go to bed when feeling sleepy (after following a wind-down routine to prepare the mind and body for sleep), if not able to sleep within approximately 15 minutes one should get out of bed, engage in relaxing activities in a different room and only return to bed when feeling sleepy. Stimulus control identifies the need for regular bed and rise times.
Sleep restriction	Sleep restriction aims to reduce sleep onset latency and waking after sleep onset by increasing the sleep drive and overcoming compensatory strategies that disrupt the association between bed and sleep. This is achieved by limiting the time in bed to the average total sleep time and then delaying the bed time. As sleep efficiency increases patients extend the sleep period in small 15-minute increments to increase overall sleep quantity.
Relaxation	Relaxation techniques (including progressive muscle relaxation and breathing exercises) are utilized to reduce physiological and emotional arousal in order to facilitate sleep.
Cognitive strategies	Cognitive restructuring and behavioural experiments may be used to test out specific beliefs related to sleeplessness. In addition, cognitive strategies might be used to reduce the impact of worry on sleep.

In our work, therapy is delivered on an individual basis in approximately eight sessions over 3 months. The intervention is written in a workbook style manual and both therapist and client have copies. The main treatment techniques utilized are based on an individual formulation of triggering and maintenance factors. The core techniques are: ensuring there is a suitable environment, stimulus control, setting regular rhythms (bed-time, rise-time and meal-times), relaxation, circadian rhythm stabilization, and increasing daytime activity. Later, the focus might include specific cognitive techniques to address fears related to sleeplessness, strategies to manage night-time worries, imagery rehearsal and grounding techniques to manage night-time awakenings/nightmares and tapering of hypnotic medication. Finally, the interaction between sleep disturbance and psychotic experiences is addressed as needed. The intervention is deliberately simplified, with the principal therapeutic technique being stimulus control: learning to associate the bed with sleep. In order to associate the bed with sleep, bed must only be used for sleeping. If a person is lying in bed awake for approximately quarter-of-an-hour, then he or she is encouraged to get up and spend time relaxing in another room before returning to bed. This means that the individual breaks the association between bed and sleeplessness and instead strengthens the association between bed and sleep. It should be noted that sleep restriction is not used within our protocol.

**Figure 1.**
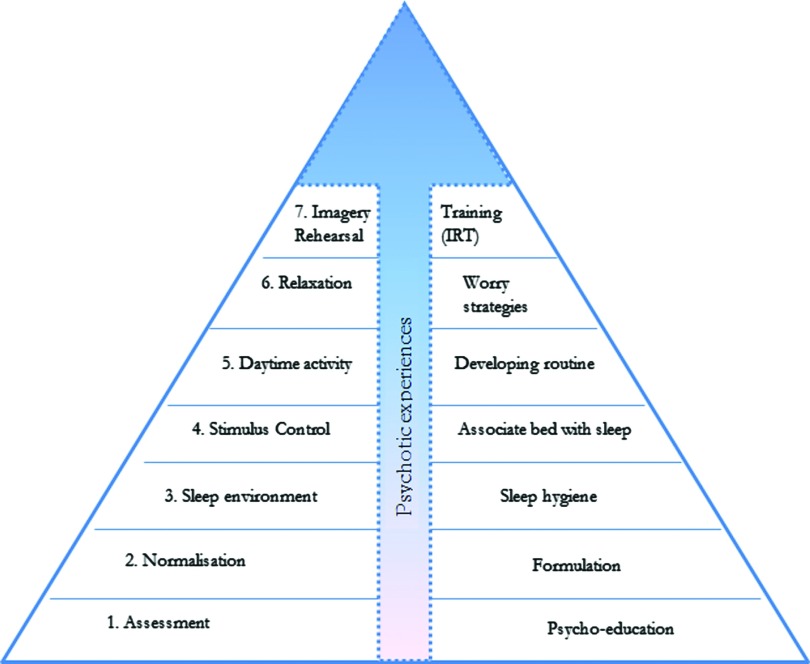
A hierarchy of treatment technique

One general issue in this work is how to approach multiple factors causing sleep disruption. For example, a patient may be having night-time panic attacks, intrusive voices, fears about their safety, and have no bed to sleep in. Put simply, where should therapy start? We work using a basic hierarchy of intervention, with the later course of therapy built on initial foundation techniques. Figure 1 displays a temporal order and hierarchy of techniques with basic sleep approaches being followed by increasingly specialist techniques. Psychotic experiences can influence all stages of the intervention, meaning slight adaptations to techniques, but the psychotic experiences are most commonly only directly addressed at the final stage. Our practice is to implement the foundation techniques, monitoring outcomes throughout, so that if improvement is not occurring then the need for additional techniques is established. Finally, a clinician should be aware that sleep disturbance may be a consequence of sleep apnoea, a sleep related breathing disorder characterized by loud snoring, periods when breathing stops during sleep and extreme daytime fatigue, which has elevated rates in schizophrenia. This would lead to the use of a distinct sleep apnoea treatment.

### Treatment adaptations

Table 2 summarizes the 12 key factors related to sleep and schizophrenia that we have identified in clinical practice, the associated treatment strategies, and their adaptations for this population. Each factor begins with a description of the problem, followed by information on the adaptation of techniques, and the differences from traditional approaches.

**Table 2.** Summary of key sleep disruption factors and associated treatment strategies

	Factors that contribute to sleep disturbance	Typical presentation for patients without schizophrenia	Typical CBT-I strategy	Adaptation of treatment for patients with schizophrenia
1	Poor sleep environment	Relatively minor environmental features e.g. temperature, light, noise.	Minor adjustments to sleep environment suggested in sleep hygiene and stimulus control	Major adjustments of sleep environment often needed e.g. buying a bed, place towels or table cloth over window to make sure they are dark at night
2	Lack of daytime activity	Concern regarding real and perceived deficits in daytime performance due to sleep problem.	Cognitive restructuring and behavioural experiments demonstrating the ability to complete daily tasks even following a night of poor sleep. Address attentional biases and misperception of effects of sleep problem.	Focus on increasing otherwise sparse daytime activity via activity scheduling, “5-ways-to-wellbeing”, using sleep diaries for activity planning and as a positive data log. Using the rationale of a 24-hour intervention to improve sleep.
3	Lack of evening activity	Not applicable	Not applicable	Developing activities in the evening to overcome phase advance or to prevent time in bed not sleeping.
4	Disrupted circadian rhythm	Circadian rhythm disruption includes phase advance, phase delay, hypersomnia and erratic sleep patterns.	Stimulus control, resetting rhythms by adjusting the sleep window in small increments (15 minutes each week) to fit the social environment.	Resetting circadian rhythm using regular routines for morning (rise-up) and evening (wind-down), setting meal-times interspersed with activities and using pleasant activities to reinforce rise-times. Testing out beliefs about increasing daytime activity to overcome hypersomnia.
5	Sleep as an escape from distressing experiences e.g. voices	Not applicable	Not applicable	Increase ability to cope with distressing voices or paranoia (drawing upon CBT for psychosis), improve use of sleep as a way of coping with voices.
6	Fear of bed	Fear of bed due to fear of sleeplessness.	Cognitive restructuring of sleep related unhelpful beliefs, attentional focus redirected from threats to sleep; stimulus control.	Fear of bed due to association of bed with distressing psychotic or traumatic experiences. Graded exposure to bed, increased coping strategies for psychotic experiences and when these are both in place, stimulus control.
7	Nightmares	Prevalence associated with posttraumatic stress disorder.	Imagery rescripting for repetitive nightmares, combined use of CBT-I and imagery rehearsal training.	Psychoeducation, grounding techniques, imagery rescripting for repetitive nightmares and positive imagery for themes of non-repetitive nightmares. Potential medication review.
8	Night-time awakenings	Tendency to recall periods of light sleep and misperception of time awake during night.	“Quarter-of-an-hour rule” to reduce time in bed not sleeping after night-time waking.	A range of unusual experiences at night are often described, including hypnogogic/hypnopompic hallucinations, nocturnal panic, and depersonalization after waking. Grounding techniques, relaxation, “quarter-of-an-hour rule”, psychoeducation, formulation, reduce arousal prior to sleep onset and after waking.
9	Sleep disrupted by voices/paranoia	Not applicable	Not applicable	CBT for psychosis techniques including increasing activities that lessen psychotic experiences, reducing triggers to psychotic experiences and challenging beliefs about power of the persecutor/voice.
10	Worry	Worry regarding sleep related threat/content of worries focused on sleep	Cognitive restructuring and behavioural experiments	Additional consideration of worries related to psychotic experiences via relapse prevention planning, indirectly testing persecutory fears by increasing daytime activity.
11	Neuroleptic medication side effects	Not applicable	Not applicable	Consideration of a range of side effects including daytime fatigue, increased risk of nightmares, sedative effects. Medication review, including consideration of timing of medications.
12	Reducing hypnotics	Tolerance and rebound effects.	Gradual withdrawal and tapering medication.	Consideration of rebound effects on psychotic experiences and tapering hypnotics in the context of other prescriptions e.g. neuroleptics.

*1. Poor sleep environment*. The environment has a major impact on promoting or disrupting sleep. Fundamental changes to the sleep environment are sometimes necessary. We find some patients don't even have a bed, sleep on the sofa, or have no blinds. Rooms may be cluttered or unclean. All of these environmental features are disruptive to sleep.

“Setting the scene for good sleep” is a simple clinician rationale shared with patients for addressing the sleep environment. The message concerns not fighting insomnia but rather facilitating the individual to ensure the environment is set up for good sleep. This is essential in order for stimulus control to be successful. For example, if an individual is using the sofa as a place to relax and watch TV, but also as a place to sleep, the individual will be unable to adhere to the principles of stimulus control (only using bed for sleep). Therefore, a practical approach is encouraged. Home visits allow direct assessment of the sleep environment and enable clinicians to offer practical support to facilitate changes (e.g. putting up a blind). It is the scale of change often needed to the sleep environment that we highlight.

Achieving an appropriate sleep environment, or good sleep hygiene, is an important foundation for improving sleep. In CBT-I this includes lifestyle (e.g. caffeine and alcohol intake) and environmental factors. The existing CBT-I literature suggests minor amendments to environment, for example adjusting the temperature of the bedroom. However, the alterations needed are often far greater for patients with schizophrenia.

*2. Lack of daytime activity*. Many patients have a lack of activity. Inactivity may be caused by sleep problems due to daytime fatigue but it also contributes to sleep disturbance due to diminished sleep drive. For example, many patients have no planned activities, meaning that they have no motivation to get out of bed in the morning and hence may sporadically nap or rest on the sofa, or even in bed. This reduces sleep drive at night-time, disrupts the association between bed and sleep, and contributes to low mood, all of which may cause sleep disturbance.

“A 24 hour problem requires a 24 hour solution” is a simple rationale shared by clinicians for increasing daytime activity. Traditional activity scheduling is supplemented with activities that ensure physical exertion to increase energy in the day and increase fatigue at night-time. The “five ways to wellbeing” concept can be useful in broadening the range of activities and enhancing psychological wellbeing (Foresight, 2008).

Light plays an important role in regulating our circadian rhythm. Natural daylight has over 10,000 lux (the measure of intensity of illumination). In comparison, artificial light in an average room has 50–500 lux. Therefore, ensuring an outdoor activity (e.g. walking to the shop) occurs each day enables both exposure to light and increased activity. The impact of exposure to light can be enhanced by the timing within the circadian pattern. For example, a walk in the morning will have a greater impact on advancing the circadian rhythm (waking earlier). Scheduling a range of activities, with consideration of their circadian timing and effects on sleep, is a foundation for nearly all patients to improve their sleep, wellbeing, and satisfaction with daytime activity.

The cognitive approach to insomnia focuses on the feared and real deficits in daytime performance and functioning as a result of sleep disturbance (Harvey, 2002). However, due to the poverty of daytime activity there are typically few feared consequences of sleeplessness on daytime performance in this psychiatric population. Instead, a pattern of fatigue and daytime napping, long lie-ins and early bedtimes - in other words hypersomnia and circadian disruption - is more likely to develop. Therefore, in our experience, attentional biases and distorted perception of performance are not necessarily required as features of the intervention.

*3. Lack of evening activity*. Often a lack of activity and social connection results in patients going to bed very early. This either results in patients lying in bed wide awake for hours as their bodies are simply not ready for sleep or if their circadian rhythm adjusts they will get used to going to bed early and then wake very early, a pattern known as an advanced phase circadian rhythm disruption (APCRD).

A rationale that we give in therapy is: “It might sound odd, but going to bed later actually means getting to sleep faster and at the time that you want. The 24-hour body clock ensures that peak alertness and performance occurs in the day and a good block of sleep occurs at night.” This identifies the need to add activities in order to delay bed-time and realign the circadian rhythm for peak daytime performance and night-time sleep. In addition, delaying bed-time can reduce sleep onset latency (SOL), the time between going to bed and going to sleep. Activity planning includes meal-times and activities prior to the initiation of the wind-down routine. The wind-down routine is a specific plan for preparing for sleep 60–90 minutes before sleep onset. It involves ending tasks for the day and engaging in relaxing activities. Once again this needs detailed planning on a practical level, for example getting a new box set of a favourite TV-series. Relaxation is often incorporated as a calming activity whilst delaying bed-time.

Existing guidance focuses on the adjustment of bedtime to address SOL and APCRD but this does not usually consider the impact of poverty of activity or social contact. Thus detailed activity scheduling of the evening period can increase the likelihood of successfully realigning the patient's circadian rhythm for presentations with APCRD.

*4. Circadian rhythm*. Erratic sleep patterns, swinging between too much and too little sleep, have been documented in people with schizophrenia (e.g. Wulff, Dijk, Middleton, Foster and Joyce, 2012). For example, the association between sleeplessness and psychosis leads some to oversleep in order to prevent relapse or cope with psychotic experiences. However, too much sleep one night makes it hard to sleep the next night due to a reduced sleep drive. This leads to a pattern of too much and too little sleep, in which the anchors of going to sleep and waking up are varied. Missed appointments can be a reminder to ask about sleep problems, as APCRD (going to sleep too early), delayed phase circadian rhythm disruption (DPCRD: going to sleep too late and waking late) and hypersomnia (oversleeping) often disrupt daytime activity. The lack of daytime activity and routine also results in a loss of social events/demands that help to synchronize the circadian rhythm (social zeitgebers). The circadian rhythm includes sleep, activity, and other biological processes such as body temperature and hormone production. Many patients have a poor and inconsistent diet, including high levels of caffeine and nicotine, which may further disrupt the body's internal clock.

We provide a clinician rationale that highlights the importance of resetting the daily rhythm, including times for sleep, rising, and meals: “We live in a world of rhythms, for example night follows day, and we need to get your rhythms back in sync”. In order to reduce the sleep pendulum swinging from too much to too little sleep, we first need to establish a regular sleep routine (bed-time and rise-time) to maintain everyday (including weekends). Detailed planning of a wind-down routine results in sleepiness at bed-time and supports stimulus control. A rise-up routine tries to ensure that light and activity enables the individual to overcome the commonly-occurring tired, groggy feeling on waking, known as sleep inertia. For many people with DPRCD or hypersomnia, the mantra is to “do one thing in the morning- open the curtains!” This one action gets the individual active, out of bed, and exposed to light. Ensuring pleasurable activities are planned for the morning (e.g. appetising breakfast, listening to a favourite CD, or meeting a friend) rewards the individual for getting up at their planned rise-time. Setting regular meal-times interspersed with planned activities establishes a rhythm to the day. Therefore, detailed planning of the whole 24-hour period may be necessary, including adjustments to address phase advance/delay.

Circadian rhythm disruption is an important category of sleep disorder. Correction can take time. It is addressed by gradually adjusting the sleep window in small increments (15 to 30 minutes each week). The erratic and excessive sleep patterns characterizing this group can be influenced by psychotic experiences; this has not been addressed previously in CBT-I.

*5. Sleep as an escape from psychotic experiences*. Sleep can provide important respite from distressing psychotic experiences. This leads some to overuse sleep as a strategy to manage distressing psychotic experiences. For example, one patient in our trial would attempt to sleep at 4 p.m. in order to escape hearing voices. But as their body wasn't ready for sleep they would lie awake for hours, with no distraction from the voices. This made it more challenging to cope with the voices, which led to frustration and despair, making it harder to sleep and perpetuating the cycle. Thus sleeping to escape psychotic experiences can have the unintended effect of unsettling sleep, by disrupting the circadian rhythm, increasing attention and intentional effort to sleep, and making it more difficult to sleep at night due to the extended SOL. For those who can get to sleep, the reliance on sleep as a coping strategy can lead to hypersomnia. Being asleep most of the day and night means missing out on opportunities to engage with the social world and increases the sense of isolation, hopelessness, fatigue, and low mood, all of which can contribute to an increase in distressing psychotic experiences. In sum, the reliance on sleep as a way to escape distressing psychotic experiences has the unintended effect of disrupting sleep, which further exacerbates psychotic experiences creating a vicious cycle of distressing experiences, disturbed sleep, and lack of coping strategies.

“There is nothing wrong with using sleep to escape distressing psychotic experiences - it's a very helpful strategy, so we want to enhance it and add in other coping strategies too”. This rationale, given by our clinicians, identifies the importance of improving sleep and increasing the range of coping strategies. Therefore two elements are involved: 1) increasing the range of coping strategies for psychotic experiences: 2) enhancing sleep to manage/reduce psychotic experiences.

First, common simple strategies from CBT for psychosis are used. These include normalizing, psychoeducation, and connecting with personal accounts of others with similar symptoms to decatastrophize the experience. For patients hearing voices, this may also include enhancing coping strategies and reviewing the relationship with the voices (e.g. recognizing voices are not a good source of information).

Second, sleep is reclaimed as a tool for escaping distressing psychotic experiences. Better quality sleep helps to reduce the frequency and intensity of distressing psychotic experiences and improves coping capacity. For example, delaying bed-time actually reduces the time listening to distressing voices without distraction. Therefore, planning relaxing, engaging, enjoyable evening activities reduces distress from voices and promotes good sleep by reducing arousal. Delaying bed-time also addresses APCRD and reduces total sleep time to overcome hypersomnia. Planning daytime activity helps reduce hypersomnia, improve sleep at night-time and increase mood. It also provides opportunities to test out beliefs about psychotic experiences.

In the existing CBT- I literature, the formulation of distressing experiences disrupting sleep has been confined to worries, unhelpful appraisals and heightened emotion. This represents the first extension of this principle to cover psychotic experiences.

*6. Fear of bed*. For some patients bed has become more than a place associated with sleeplessness: it is also associated with intrusive memories of traumatic events including distressing psychotic experiences. Bed has become a place that is feared and avoided. Avoiding bed means disrupted sleep, either due to avoidance of sleep or poor sleep hygiene.

“Bed equals sleep” is the mantra of stimulus control. This technique is based on the core principle of associating bed with sleep. However, before bed can be re-associated with sleep it needs to be experienced as a safe place. For example, one patient in the trial feared sleeping in their bed as they believed they would be overwhelmed by hearing distressing voices and re-admitted to a psychiatric ward. If the “quarter-of-an-hour rule” is employed at the first stage, anxiety will be exacerbated as the individual will leave the bedroom prior to anxiety habituation. In short, the “quarter-of-an-hour rule” could serve as an avoidance strategy that would increase anxiety about being in bed. Therefore, prior to stimulus control strategies being initiated, an exposure hierarchy is developed and graded exposure to being in the bedroom is completed. In order to complete the hierarchy, coping strategies for managing voices must be enhanced, for example, having a radio in the bedroom. When the individual no longer feels anxious about being in the bedroom and has learnt that they can cope with hearing voices in the bedroom just as well as they can cope in other places, stimulus control strategies are used.

Fear of bed, due to a fear of sleeplessness is included in the cognitive perspective on insomnia (Harvey, 2002) and in hyperarousal models of insomnia (Espie, Broomfield, MacMahon, MacPhee and Taylor, 2006). In these models, the anxiety response/hyperarousal relates to a negative cognition of the feared consequences of loss of sleep and the subsequent conditioning effects of this arousal. Therefore cognitive restructuring to explore the belief about the consequences of sleeplessness and stimulus control strategies are engaged at the outset. This is in direct contrast to the consideration that stimulus control could exacerbate anxiety if it becomes an avoidance that prevents addressing an alternative underlying fear. This is demonstrated in our example in which the patient was fearful of being overwhelmed by voices rather than being fearful of sleeplessness. Therefore, identifying the underlying cognition that leads to a fear of bed is critical in selecting the appropriate intervention techniques.

*7. Nightmares*. Vivid, disturbing dreams leading to daytime preoccupation and impaired functioning are frequently reported by patients with schizophrenia (Sheaves, Onwumere, Keen, Stahl and Kuipers, 2014). For example, dreams may be of seeing the mutilated body of a family member or being kidnapped.

“Feeling calm and safe makes us less likely to have nightmares” is a rationale given by the clinician to work on reducing the likelihood of nightmares occurring by ensuring the foundations for good sleep are established, thus reducing arousal prior to sleep onset. This process includes psychoeducation about sleep stages, especially Rapid Eye Movement (REM) sleep, as nightmares often occur during REM sleep. The proportion of REM sleep increases throughout the sleep-period; therefore there is greater opportunity for nightmares if one has hypersomnia. Increased REM sleep and nightmares specifically may be a side-effect of medication (e.g. certain antidepressants). Therefore reducing REM sleep, by sleeping for a shorter period or changing medication (after discussion with the prescriber), reduces the opportunity for nightmares to occur. Second, grounding techniques, based on post-traumatic stress disorder treatment protocols are used to reduce distress on waking.

An example of the rationale used to experiment with imagery is: “Imagination is very powerful - just look at the way nightmares leave you feeling and they are all a product of your mind. Therefore, let's use our powerful imagination to make you feel safe instead of scared”. For repetitive nightmares standard imagery rehearsal training protocols are utilized.

*8. Night-time awakenings*. A range of unusual experiences at night are described by patients with schizophrenia; these include hypnogogic/hypnopompic hallucinations, nocturnal panic, and depersonalization after waking. For example, one patient in the trial described waking suddenly, smelling burning, and thinking she was in a war zone. As she had never been to war, she had thoughts that her life was not her own and that perhaps she was a different person altogether.

“It's common to wake in the night, it's pretty common to wake with a jolt too” is a rationale used by clinicians to highlight the normal sleep cycle includes brief awakenings every 60–90 minutes. Specific psychoeducation concerning hypnogogic and hypnopompic hallucinations is used to normalize, decatastrophize, and formulate the response. This reduces the fear of night-time experiences and subsequently reduces arousal. On waking, the aim is to orient the patient in time and place, thereby reducing distress and confusion. Cue cards with grounding or reassuring statements are placed near to the bed to ensure quick and easy access. Relaxation is used to reduce arousal and then the “quarter-of-an-hour rule” is applied to ensure that the bed does not become associated with distressing night-time wakefulness.

Hypnogogic and hypnopompic hallucinations often occur as part of other sleep disorders, especially narcolepsy, or as a feature of psychosis. Therefore traditional treatment focuses on dealing with the underlying cause. However, in our work we also focus on developing coping techniques (e.g. grounding, relaxation) and a clear understanding (e.g. psychoeducation, formulation) to reduce the fear associated with the experience. This in turn is likely to reduce arousal and promote good sleep.

*9. Sleep disrupted by voices/paranoia*. Psychotic experiences can directly disrupt sleep. For example, voices may threaten to harm the patient if he or she sleeps. The patient may then obey the voices, trying to avoid or restrict sleep, thus leading to hyperarousal, delayed sleep onset, and fragmented sleep. Alternatively, voices commanding the patient to sleep at certain hours can result in APCRD.

“If it's stopping you sleep we’ll work on it” is a simple clinician rationale for directly addressing psychotic experiences within a sleep intervention. Understanding how psychotic experiences influence sleep is an important first step, achieved by developing maintenance cycles of sleep disturbance and psychotic experiences. The key techniques (stimulus control, daytime activity, relaxation, and worry strategies) are used as a foundation for improving sleep. Once these are in place, specific techniques to address psychotic experiences may be used. Therefore the work may include an amalgamation of CBT-I and psychological strategies for psychosis, used in tandem and sequentially to maximize benefits depending on the case conceptualization. Working with hallucinations may include: 1) discussing personal accounts from others in order to normalize the experience and think about alternative ways of reacting; 2) identifying triggers for voices (e.g. inactivity, low mood, and anxiety) and then directly reducing theses triggers; 3) diverting attention away from voices by addressing beliefs about the need to attend to the content of voices; 4) evaluating beliefs about the power and omnipotence of the voices by adjusting sleep habits (e.g. getting up for one minute when the voices command the individual to lie in bed); 5) adapting worry period strategies to gain control over the experience of voice hearing; 6) addressing reactions to the voices, for example depression, anxiety or anger, and developing a less emotionally aroused response to the voices.

Working with paranoia in this context may include: 1) normalizing and decatastrophizing the occurrence of such fears; 2) identifying and reducing triggers (typically anxiety, depression, worry, and inactivity); 3) reviewing sleep-related fearful beliefs (e.g. that the person will be attacked during the night). In the later stages of the sleep intervention, if sleep is still not improving it may be indicated for a small number of patients to reduce daytime arousal by testing out paranoid fear beliefs directly (i.e. in these instances the daytime paranoia is driving the poor sleep). This has not been used in our trial but in our wider clinical experience is very occasionally used.

*10. Worry*. Worry is a major cause of sleep disturbance. However, the specific content of worry tends to be somewhat distinct in schizophrenia, for example worries that others are plotting to cause harm. Worries regarding the negative impact of sleeplessness often relate to fears of relapse or not being able to cope with psychotic experiences. In sum, worry typically relates to psychotic experiences rather than daytime effects of sleeplessness.

A rationale shared by the clinician to highlight the way worry, and associated hyperarousal, disrupts sleep is: “Worry takes us to the worst-case-scenario in our mind, this triggers anxiety and our body reacts by being on ‘high-alert’ this is the opposite to the relaxed, calm feelings we need in order to sleep. We need to feel safe to fall asleep”. Our group has demonstrated the effectiveness of worry intervention techniques (e.g. worry periods) in reducing worry and reducing persecutory fears (Freeman, Dunn et al., 2015). Thus established worry strategies, which focus on the process rather than content of worry, are used.

Additional consideration may be needed for specific worries relating to psychotic experiences and fear of relapse. A review of past relapses, evidence of new awareness and improved coping, and a relapse prevention plan are used to help reduce such fears.

*11. Neuroleptic medication side-effects*. Fatigue and lack of energy are common features for people with sleep problems and patients with schizophrenia. For example, a patient in the trial struggled to stay awake in session despite a cup of tea and adequate room lights. Due to the sedative effects of neuroleptic medication, daytime fatigue in patients with schizophrenia is commonly attributed to medication side-effects.

“Let's look at all the possible things that could be leading to you feeling so tired, including your medication, that way we’ve got the best chance of working out what to change” is a rationale shared by the clinician for understanding the prescribed medication and potential side-effects (e.g. fatigue, anxiety and nightmares). Reviewing a timeline of medication changes and symptom onset can help to identify any correlations. Medication should be reviewed in collaboration with the prescriber. However, psychological strategies can be of additional benefit, for example scheduling pleasant morning activities as part of a rise-up routine is particularly important for people who report a “hangover” effect from medication. Behavioural experiments can be used, for example, to test beliefs about the production and use of energy or about the effects of caffeine reduction, including concerns about the taste of decaffeinated drinks.

*12. Reducing hypnotics*. Hypnotics are commonly prescribed in addition to neuroleptic medication. However, tolerance and rebound effects of hypnotics are well established. For example, a patient seen in the trial took hypnotics in the early afternoon for fear that lack of sleep would lead to relapse. However, additional hypnotic medication was then taken later in order to try to get night-time sleep, further increasing tolerance to the drug.

“Short term pain for long term gain” is a colloquial phrase employed in the clinical work to emphasize that reducing hypnotics is likely to lead to rebound effects in the short-term but considerable sleep improvements in the long-term. Tapering and stopping hypnotics needs to be considered in the context of neuroleptic and other medication. Discussing the potential negative effects of hypnotics may open a wider conversation about other medication and collaboration with the prescriber is essential. Adjusting timings and dosage can be important. Psychoeducation regarding rebound effects helps manage expectations and gives a clear rationale for tapering. Gradual exposure, cognitive restructuring, behavioural experiments and psychological strategies for psychosis can also be used to tackle fears of increased psychotic experience to enable tapering. The reduction of hypnotics can be facilitated by CBT-I; however, in this population consideration of rebound effects exacerbating psychotic experiences and tapering hypnotics in the context of neuroleptic medication are novel features.

## Conclusion

There is increasing recognition of the prevalence and severity of sleep problems in patients with schizophrenia. There has, however, been far less discussion of the clinical treatment. We have highlighted adaptations to existing treatment, organized around 12 factors, which we think disrupt sleep in patients with schizophrenia. It is hoped that this paper will encourage and assist others to use evidence-based psychological approaches for sleep problems in schizophrenia. We expect increased discussion and sharing of ideas as this novel field develops.
